# Application of Equilibrium Models of Solution Hybridization to Microarray Design and Analysis

**DOI:** 10.1371/journal.pone.0011048

**Published:** 2010-06-10

**Authors:** Raad Z. Gharaibeh, Joshua M. Newton, Jennifer W. Weller, Cynthia J. Gibas

**Affiliations:** 1 Department of Bioinformatics and Genomics, The University of North Carolina at Charlotte, Charlotte, North Carolina, United States of America; 2 Department of Physician Assistant Studies, Wake Forest University School of Medicine, Winston-Salem, North Carolina, United States of America; University of Minnesota, United States of America

## Abstract

**Background:**

The probe percent bound value, calculated using multi-state equilibrium models of solution hybridization, is shown to be useful in understanding the hybridization behavior of microarray probes having 50 nucleotides, with and without mismatches. These longer oligonucleotides are in widespread use on microarrays, but there are few controlled studies of their interactions with mismatched targets compared to 25-mer based platforms.

**Principal Findings:**

50-mer oligonucleotides with centrally placed single, double and triple mismatches were spotted on an array. Over a range of target concentrations it was possible to discriminate binding to perfect matches and mismatches, and the type of mismatch could be predicted accurately in the concentration midrange (100 pM to 200 pM) using solution hybridization modeling methods. These results have implications for microarray design, optimization and analysis methods.

**Conclusions:**

Our results highlight the importance of incorporating biophysical factors in both the design and the analysis of microarrays. Use of the probe “percent bound” value predicted by equilibrium models of hybridization is confirmed to be important for predicting and interpreting the behavior of long oligonucleotide arrays, as has been shown for short oligonucleotide arrays.

## Introduction

DNA microarrays [1] have revolutionized every area in biology [2]. Microarrays allow thousands of genes to be assayed at once, offering global views of biological processes at the transcriptional level [3], as well as allowing surveys of DNA sequence variation [2], and alternative splicing [4]. Integration of the results with other data informs many projects, such as those that perform cancer classification [5], genome annotation [6] and functional genomics [7]. The biology research community has invested heavily in microarray technology and values it, despite ongoing challenges with data quality and data interpretation.

DNA microarray chips are constructed on a solid surface, which provides reactive groups to which can be attached nucleic acids, called *probes* [8]. The sequences of these probes are designed to interact with selected mixtures of labeled nucleic acids, called *targets* [8]. While PCR preparation is used occasionally, most probes are synthesized using organic chemistry methods. Lengths vary from short (20–30mer) to long (50–70mer) sequences [9], and often include a carbon-spacer with an amino or thiol reactive group on one end for covalent surface attachment. The origin of the targets depends on the type of the experiment, ranging from mRNA for gene expression studies to genomic DNA for SNP and CNV studies. A stable target-probe heteroduplex produces a detectable signal, whose interpretation depends on the experimental details, such as how target processing steps affect transcript abundance in gene expression experiments [10], [11], and whether probe sequences match sample SNP alleles [12].

Probes in the length range 50–70 nucleotides deliver higher sensitivity than shorter probes [9], [13], due to their higher target affinities. Chou et al. [14] found that 60-mer probes can detect targets with eightfold higher sensitivity than 25-mer probes. Thus, depending on the question, microarrays using longer oligonucleotides will require fewer probes [15], [16], and uniqueness can be more easily achieved. While sensitive and specific in gene expression experiments, long probes have disadvantages when microarrays are used for other applications. The usability of long probes in SNP detection, for example, is limited, because a target containing a single mismatch can still bind to the probe with an affinity nearly as high as the perfect match. Long probes are rarely used for detecting splice junctions by spanning two exons, for similar reasons. Since sequence specificity decreases as the length of probes increases, the excellent sensitivity of the long probe has to be balanced with concerns about cross-hybridization with unintended targets of high affinity, and with the potential for stable structure formation in the probe. Long probes are also difficult to optimize in situations where the target sequence is very short.

From the analysis perspective, however, long probe platforms are simpler to deal with than platforms using multiple short probes. An averaging method is usually not needed, since there is often only one probe interrogating each gene. Short probe platforms require an averaging or summarization of a set of probes for each gene. Handling noise and specificity factors is much more complicated on short probe platforms due to the compact nature of these arrays, and to the higher noise generated by short probes [9].

These considerations, along with the decreasing cost of synthesis, have led to widespread use of long oligo microarrays [17], especially when arrays are being designed for non-model organisms where a commercial short-oligo array is not readily available. Despite the popularity of long-oligo platforms, model development for the interaction of the reactants on long-oligo arrays has received relatively little attention. Partly because the interactions fit a two-state hybridization model, most biophysical studies have focused on short oligonucleotide microarrays [18], [19], [20]. Our long-term goal is to address the deficiencies in modeling of long-oligo microarrays, and here we report a significant step towards that goal.

### The effects of mismatches in the duplex on hybridization

The signal from a spot is used as a proxy for the amount of target present, frequently in a ratio relative to the same target in another sample. Accurate interpretation of the signal relies on the specificity of the hybridization reaction, and whether conditions allow discrimination between fully complementary hybrids and those with some degree of mismatch [21]. Short oligonucleotide probes are well suited to discriminating small sequence differences and the effect of sequence variation on their hybrid stability has been thoroughly studied [22], [23], [24], [25], [26]. While the biophysics of short oligonucleotide binding in solution is well understood [27], the binding properties of longer, tethered oligonucleotides are less well characterized [27], although it is still the case that the effects of mismatches depend on position and context, unlike the case of very long polymers.

The degradation in hybrid stability when probes and targets are mismatched can lead to a number of false conclusions. The total signal per mass of target is decreased when a mismatch is present, and even analyses that rely on relative signal between two channels will be subject to errors as the signal-to-noise ratio decreases. Not all cross-hybridization scans take mismatches into account, which can lead to false positive results at different loci [28]. On the other hand, tolerance for, and correct interpretation of, mismatches allows applications such as inter-specific interrogation [29] to be successful.

A number of investigations have explored the minimum number of base pairs required for formation of a duplex to produce a signal, under given hybridization conditions, in order to determine the limits of non-specific hybridization [28], [30]. Because of the number of permutations involved, the published studies of long oligonucleotide probes use a very limited number of sequences, consider only greater than three mismatches, or use a limited target concentration range [18], [28]. By introducing mismatch permutations into the probe set rather than the target set, we were able to examine the distribution of signal across a range of interactions with more accuracy.

### Applying solution hybridization models to microarray hybridization

For long-oligo microarrays, multi-state hybridization models must be used, that take into account factors such as probe folding, target folding, probe-probe interactions, target-target interactions, and competition between closely similar sequences [31], [32], [33].

Thermodynamic models have been used to compare solution hybridization free energy (nearest neighbor) parameters to surface-solution hybridization free energy parameters [34], [35], [36]. From these studies we are able to determine which parameters are unchanged between solution and surface-solution reactions, and which must be modified.

Application of the nearest-neighbor based thermodynamic model in order to predict long oligonucleotide to target hybrid stability has been limited. In solution, the model is most accurate for probes with length ≤40 oligonucleotides [37], [38] and probes greater than 40 oligonucleotides in length have thus been considered non-ideal for nearest-neighbor based thermodynamic modeling. Several groups have shown that solution hybridization parameters based on the nearest-neighbor model can be applied to short surface-bound oligonucleotides [34], [36]. Hooyberghs et al. [35] showed that the nearest-neighbor parameters of solution hybridization and microarray hybridization are well correlated (r = 0.839) for probes of 30 oligonucleotides in length.

We have identified no studies that have applied the multi-state solution hybridization models to oligonucleotide probes longer than 40nt. In this study, we designed and modeled the binding behavior of ten sets of 50-mer probes, each set having six centrally located sequence variants with one, two or three mismatches. By creating mismatches as permutations of the surface-bound probes, rather than permutations in the target, we are able to unambiguously separate and directly compare the signal from a perfectly matched duplex and several variants. Targets have been chemically synthesized and are end-labeled, so the complexity of the solution interactions is controlled. Results indicate that current computational models of solution hybridization are effective for 50-mers across a range of concentrations. An accurate prediction of input target concentration is obtained using a ‘probe percent bound’ value, calculated using a multi-state equilibrium model of solution hybridization that is implemented in the OMP (Oligonucleotide Modeling Platform) software [39].

## Results

### Effect of central mismatches on signal intensity

**Figure 1:**
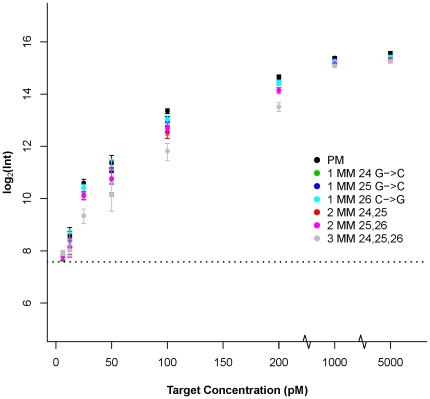
PM and MM probes responses to eight target concentrations. Hybridization signal intensities for PM probe 5005 (black) and its set of six MM variants [5035 (green), 5036 (blue), 5037 (cyan), 5038 (red), 5039 (pink), 5040 (gray)] for the eight target concentrations. The type, identity and location of the mismatch are indicated beside the symbol of each probe: 1MM: single-MM, 2MM: double-MM, and 3MM: triple-MM. The dotted line indicates the average background signal across the eight concentrations. The responses of the nine other probe sets were similar. Error bars are standard deviations.

Depending on fractional presence and context, a small number of mismatches reduces, but need not abolish, hybridization efficiency [40]. The experiments reported here were designed to determine the effect of one, two or three centrally placed mismatches in a 50-mer probe on target binding, at different target concentrations. In Figure 1, we show the effect of these mismatches (MM) on the hybridization signal intensity at eight different target concentrations, for the probe set labeled 5005. Even three mismatches did not abolish the hybridization signal. There is a target concentration effect: at the lowest target concentration examined (6.25 pM) the three categories of MMs have approximately the same signal intensity as the perfect match (PM) probe, but all of them are only marginally above the background level. At medium target concentrations (12.5–200 pM), the effect of having different numbers of mismatches is clear. As expected, the signal intensity decreases with increasing number of mismatches (PM>single-MM>double-MM>triple-MM). At higher target concentrations (1000 pM and 5000 pM) the signal intensities from each MM probe are close to that of the PM probe, and the presence of MM probes does not degrade the target concentration estimate provided by the PM probe. Similar trends were observed for the other nine probe sets (data not shown).

**Figure 2:**
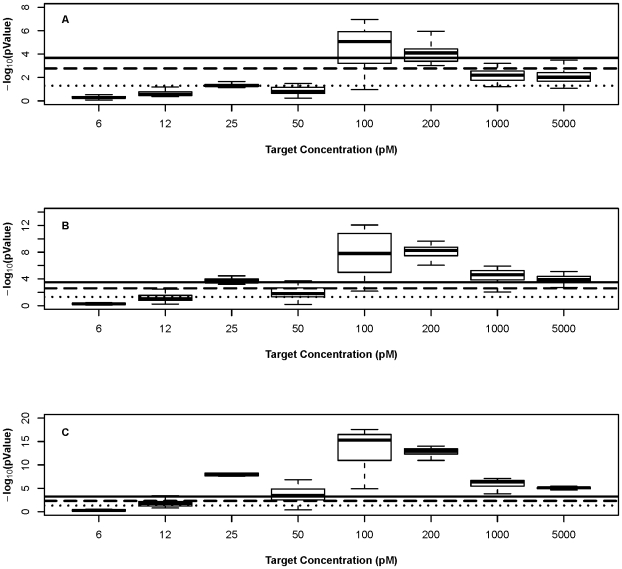
Single-MM, double-MM and triple-MM signal can be differentiated from the PM signal. Box plots for the *p*-values after testing the null hypothesis (A) PM signal intensity<single-MM signal intensity, (B) PM signal intensity<double-MM signal intensity and (C) PM signal intensity<triple-MM signal intensity for all ten sets of probes at all concentrations. The dotted line indicates *P* = 0.05 level, the dashed line indicates *P* = 0.05/(n), where n is the Bonferroni correction level, the number of hypothesis tests conducted at each concentration; 30 comparisons for single-MM probes, 20 comparisons for double-MM probes, 10 comparisons for triple-MM probes. The solid line indicates *P* = 0.05/(n), where n is the Bonferroni correction level using the sum of all tests conducted; 8 concentrations×30 comparisons for single-MM probes, 8 concentrations×20 comparisons for double-MM probes, 8 concentrations×10 comparisons for triple-MM probes. All the tests were done using a one-sided *t*-test.

We next tested whether the signal intensity differences within a related probe set, in the intermediate concentration range corresponding to the linear range of the experiment, were statistically significant. Using a one-sided *t-test*, we tested the null hypothesis that the mean signal intensity of each PM probe is lower than the mean signal intensity of its: (A) single-MM counterparts (each PM probe has three single-MM probes), (B) double-MM counterparts (each PM probe has two double-MM probes), (C) triple-MM counterpart (each PM probe has one triple-MM probe) at different *α* levels (see Figure 2).

The result of this analysis, for all of the probes, is presented in Figure 2. In all cases, the more mismatches present the larger the signal difference and the more significant that difference. Target concentrations in the range of 100–200pM yielded the most significant differences, as expected. Figure 2A shows box plots of *p*-values for the difference between PM and single-MM probes. The 100-200pM range, the signal differences between PM and single-MM probes are small but significant. Figures 2B and 2C show box plots of *p*-values for the difference between PM and double- and triple-MM probes, respectively. We were able to differentiate between PM and double- and triple-MM signals except at 6.25, 12.5 and 50pM (for PM versus double-MM) and 6.25 and 12.5pM (PM versus triple-MM).

Similar results were obtained when comparing the signals from probes with the mismatch at different positions to one another (data not shown). At target concentrations of 100 and 200pM differences were most significant, while at low end (concentrations of 6.25 and 12.5pM) the mean signal intensity differences were not significant. For none of the comparisons did the single-MM position affect the mean signal difference, nor could the double-MM comparisons discriminate the pairs, for probes in any of the sets (data not shown).

All 70 probes were classified according to the mismatch base change, and the mean signal intensities were analyzed for correlations to type and position. None of the changes (A→T, T→A, G→C or C→G) gave a consistently different response. This differs from effects seen with 25-mers, where responses showed strong sequence dependence for position and base identity of mismatches [41], [42], [43], [44], but it is consistent with the general trend that as polynucleotides get longer the sensitivity to small sequence differences diminishes. Similar results were seen for double-MM probes.

### 50-mer probe signal intensities show nonlinear response over target concentrations

**Figure 3:**
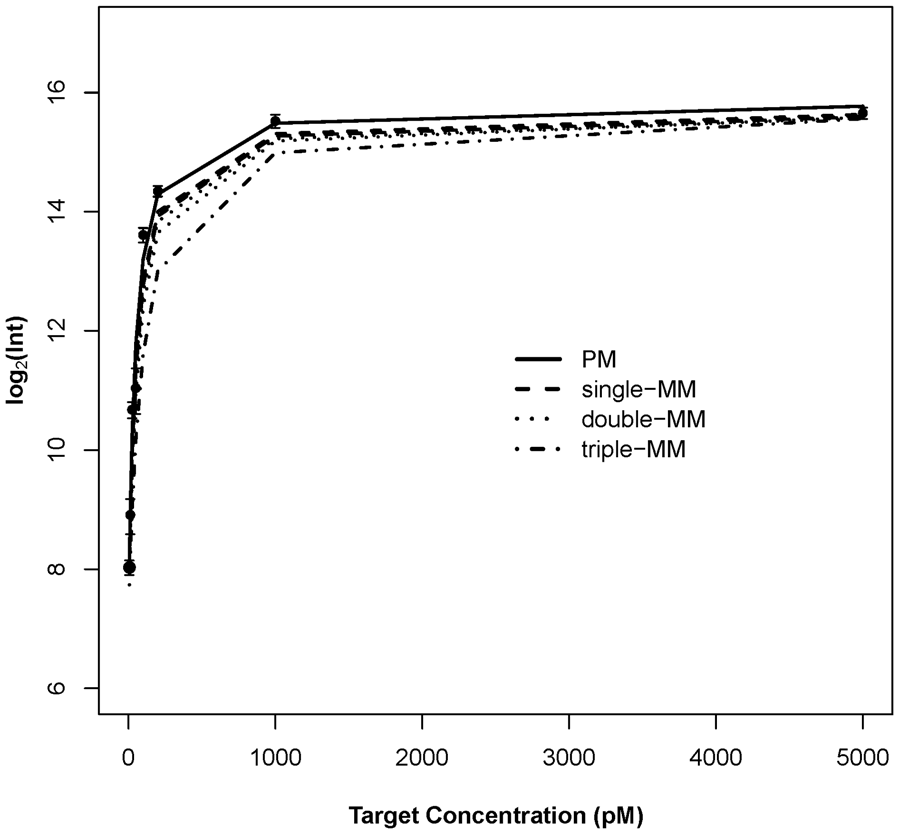
Signal intensity versus target concentration. Signal intensity versus target concentration for PM probe 5003 and its six MM variants. Points represent the observed PM intensities and lines represent Langmuir Fit model output (equation 1) for PM (solid), single-MM (dashed), double-MM (dotted) and triple-MM (dot-dashed) probes. The observed MM intensities are omitted in the figure, for clarity. Similar responses were found for the other nine sets of probes. Error bars are standard deviations.

To study the hybridization characteristics of 50-mer probes, we fitted the data to equation 1 (see Materials and Methods). Figure 3 shows, as an example, the responses at probe 5003 and its six MM probes over eight different target concentrations. The response curves closely follow the Langmuir isotherm model; the model captures the observations with an *R^2^*≥0.97. Figure 3 also shows the clear separation in the responses of a perfect match probe and its related single-, double- and triple-MM probes; again, the location and base identify of mismatches in a class are indistinguishable.

**Figure 4:**
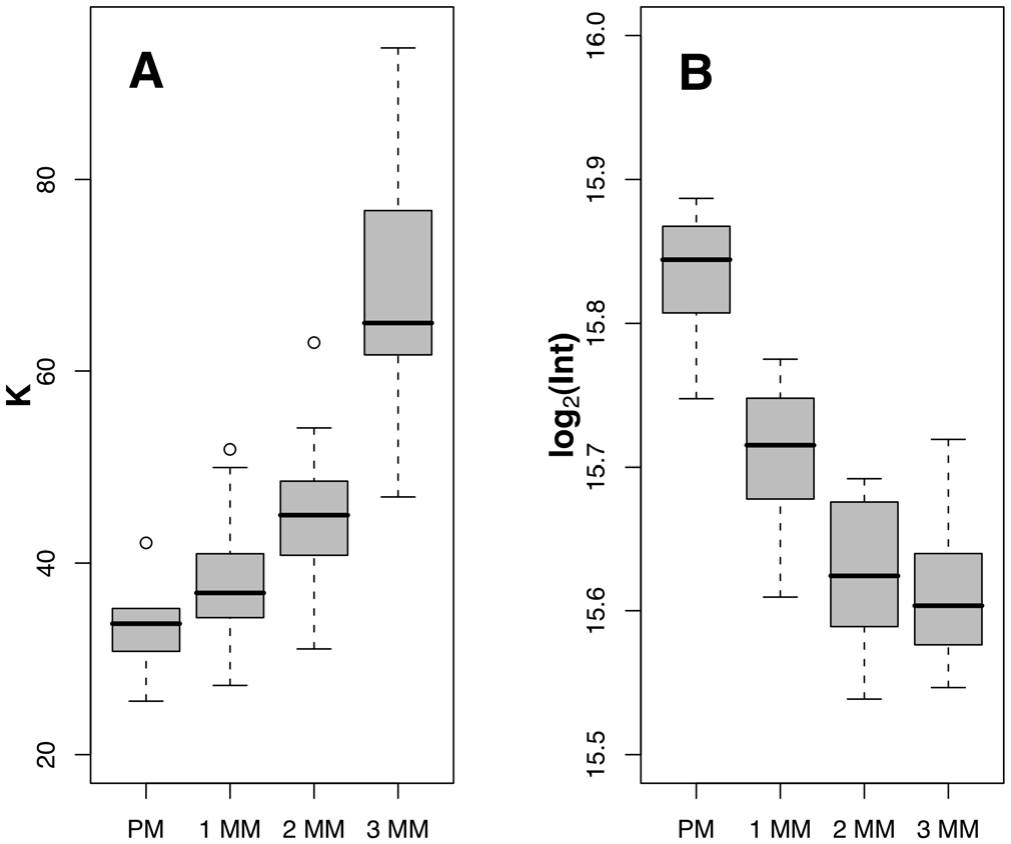
Langmuir parameters comparison. Box plots of the fitted Langmuir parameters (A) affinity constant (the fitted parameter *K* in equation 1) and (B) intensity plateau (the fitted parameters *A*+*bg* in equation 1). PM: Perfect Match, 1MM: single-MM, 2MM: double-MM, and 3MM: triple-MM.

Figure 3 also shows that the response, of all probes in all classes (PM and MM), plateaus when the target is present at 1000–5000 pM, although the intensity at which the plateaus occur differs. The relative positions of the plateaus follow the expected pattern, with the intensity of PM probe>single-MM set>double-MM set>triple-MM probe. The occurrence of a plateau does not reflect a scanner limitation. Figure 4A shows the affinity constant *K* for four groups of probes. A small *K* value corresponds to high probe affinity. Clearly the number of mismatches affects the affinity of the probe: PM probes have the smallest *K* values, single-MM probes have slightly larger *K* values, the difference is statistically significant (*P*<0.05, one-sided Wilcoxon test). The double-MM probes show significantly larger *K* values than the single-MM probes (*P*<2×10^−3^, one-sided Wilcoxon test), and the triple-MM probes are the largest (*P*<5×10^−4^, one-sided Wilcoxon test), following the expected sequence-based affinity pattern of target to probes. The intensity plateau trend is highlighted in Figure 4B: PM probes show significantly higher saturation intensities than the MM probes (*P*<3×10^−5^, one-sided Wilcoxon test), with plateau levels decreasing as mismatches increase (double-MM probes have *P*<4×10^−5^, and triple-MM probes have *P*<5×10^−4^).

### Relationship between signal intensity and *ΔG*

The results shown so far suggest the presence of detectable and significant differences between four affinity classes of probes. We next test a number of physical factors that could contribute to the observations. The first factor is the free energy of probe-target duplex formation (*ΔG_duplex_*). *ΔG_duplex_* has been invoked in several studies to explain response differences observed among short oligonucleotide probes [19], [45]. Since *ΔG_duplex_* can be easily calculated from probe sequence [27] it is one of the most frequently used screening parameters in microarray probe design [9].

Having obtained the relative signal intensity of the three MM probe classes to the PM probes, we tested whether there was a correlation between the *ΔG_duplex_* and the variance in signal intensities within a probe set and the response levels over the target concentration series, following the method of He *et al* [28]. The *ΔG_duplex_* is calculated based on the probe sequence, using the nearest neighbor method as implemented in the OMP software [27]. Duplexes containing a single MM had nearest-neighbor *ΔG_duplex_* 5% less than the corresponding perfect match duplex, double mismatch had *ΔG_duplex_* 12% less than the PM duplex, and triple mismatches had *ΔG_duplex_* reduced by 16%. The PM∶MM relative signal intensities for the thee MM classes (aggregated over all ten probe sets by class) across the eight target concentrations were: 85%, 70% and 48%, for single-MM, double-MM and triple-MM respectively. Similarly the values of predicted *ΔG_MM_*∶*ΔG_PM_* across the eight target concentrations (aggregated for the ten probe sets) were: 95%, 88% and 84%, for single-MM, double-MM and triple-MM, respectively. We related relative intensities at each target concentration to *ΔG*, and found them to be in agreement with the values reported above.

**Figure 5:**
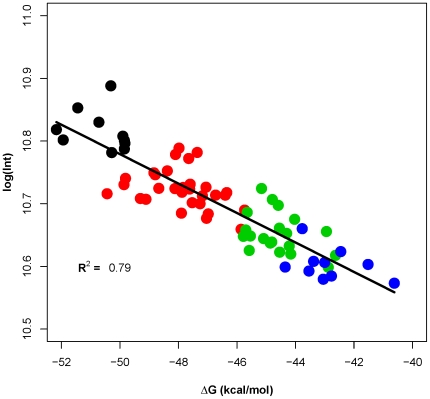
Relationship between probe-target *ΔG* and signal intensity. Relationship between (probe+target) free energy of duplex formation (*ΔG_duplex_*) and hybridization signal intensity, at a target concentration of 5000 pM. Each dot represents one probe: PM probes are shown in black, single-MM probes are shown in red, double-MM probes are shown in green and triple-MM probes are shown in blue. Similar results were seen at the other seven [target].

A model parameterized with *ΔG_duplex_* alone did not explain all of the intensity variation seen at each separate target concentration. At high concentrations of probe, the *ΔG_duplex_* changes very little since the probe concentration is constant and in >10-fold excess to target even at the highest target concentration. Contribution to the variance in signal intensity at each separate target concentration explained by *ΔG_duplex_* was explored using the simple linear model presented in equation 2 (see Materials and Methods), replacing *%Bound* with *ΔG_duplex_*. Examining the correlation between *log* signal intensity at each target concentration and the *ΔG_duplex_* reveals a weak positive correlation (*R^2^* between 0.05–0.28) at the first seven concentrations, and a stronger correlation (*R^2^* = 0.79) for the highest target concentration (5000 pM) (Fig. 5). This outcome suggests that, in principle, *ΔG_duplex_* reflects some, but not all, of the physical factors influencing signal variation.

### Explaining probe signal intensity variation using probe percent bound

We next consider the extent to which the probe percent bound (PPB) accounts for the relative signal variation between probe classes over different target concentrations. PPB reflects a multi-state equilibrium model for complex solutions, where each nucleic acid molecule is considered with respect to the entire system of possible binding partners, under the prevailing hybridization conditions (see [39] for more details). PPB can be defined, in microarray terms, as the percentage of each probe molecule that exists as a heterodimer with its target under given hybridization conditions. A PPB value of 100 indicates almost complete hybridization, while a value close to zero indicates no hybridization. Since competing reactions are taken into account, target concentrations may make significant contributions to this factor. PPB can be calculated using a standard computational method for equilibrium modeling of systems of interacting oligonucleotides (OMP for example) for each probe and target set.

**Figure 6:**
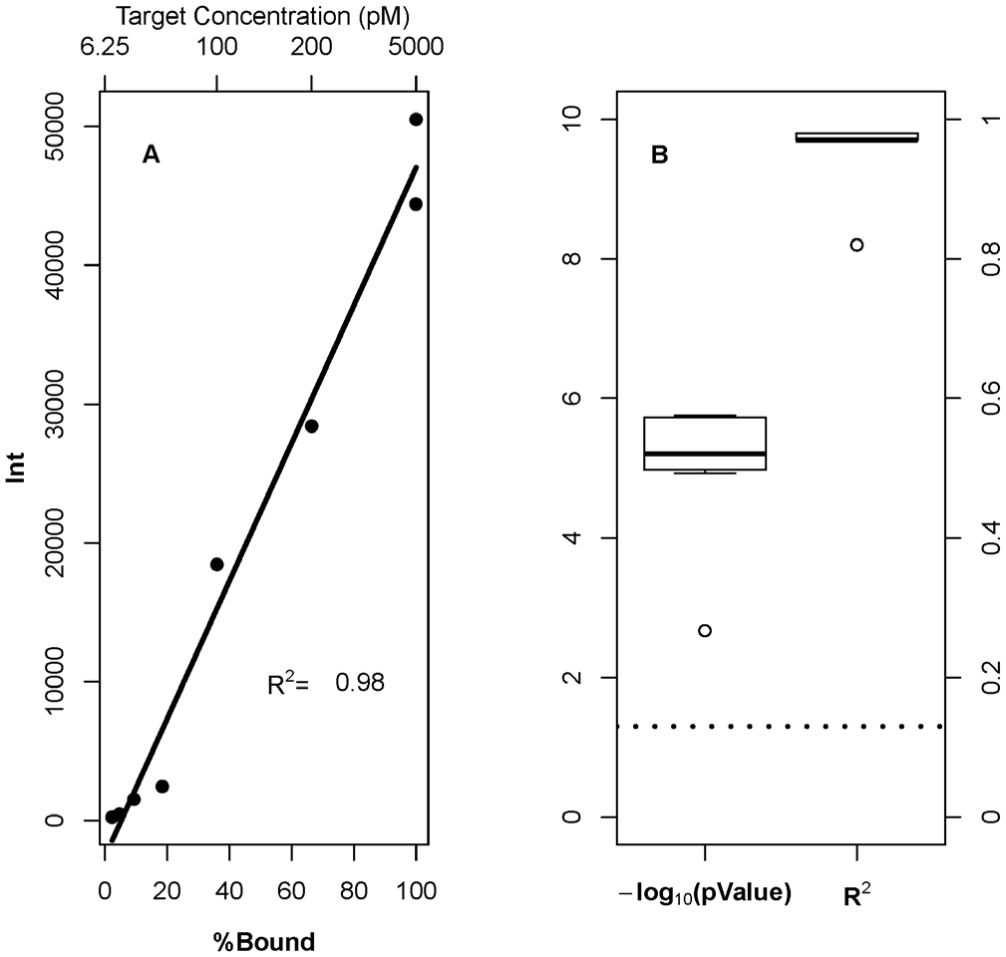
PPB is a sensitive predictor for both probe behavior and signal intensity. (A) Relationship between probe signal intensity and predicted percent bound (PPB), at each target concentration, for probe 5006 from the PM group. Points represent observed intensities, and the solid line represents the fit of the model (equation 2). (B) Box plots for the obtained *R^2^* and *p*-values of the null hypothesis test, that the *B_1_* parameter in equation 2 is equal to zero from all PM probes. Dotted line indicates *P* = 0.05 level.

We took a simple linear model (equation 2), and used as input the observed signal intensity and the probe's PPB value. Figure 6 shows a typical example of the results obtained for the PM probes after the fit. Figure 6A shows PPB vs. plateau intensity, for the representative probe 5006, and Figure 6B shows a summary of *R^2^* for the fitted model (equation 2) and the *p*-value given the test of the null hypothesis that the *B*_1_ parameter in equation 2 is equal to zero for all the ten PM probes.

Clearly, PPB explains more of the variation in signal intensity due to target concentration changes than does *ΔG_duplex_*. Fitting the data to the model (equation 2), we found a very strong correlation between PPB and signal intensity (*R^2^* = 0.98) across all target concentrations, not just the highest concentration. A coefficient this large suggests that the variation in signal intensity can be explained by PPB alone, although our test set is admittedly small. The null hypothesis, that the *B_1_* parameter in equation 2 is equal to zero, is rejected with high confidence. The same analysis was performed on each of the mismatch probe groups. The results for single-MM probes are presented in Figure S2. As in the case of PM probes, we see excellent correlation between PPB and signal intensity. The analyses of double-MM probes and triple-MM probes produce similar results and are shown in Figures S3 and S4, respectively. Combined data from all probes was also fitted to the model presented in equation 2 and the result shows that PPB explains the variation in the combined data set across all target concentrations with *R^2^* = 0.896 (Figure S5).

Examining the relationship between PM and MM PPB values reveals, as expected, a correlation between each PM PPB and its six MM PPB values (Figures S6, S7). As with the signal intensity obtained from these probes, computed PM PPB can be distinguished from MM PPB in the intermediate (linear) range (Figures S6, S7). This is consistent with the behavior of shorter oligos on SNP arrays, where differentiating SNPs is possible only if the signal is in the linear range.

## Discussion

In this study, we measured the response of targets binding to long oligonucleotide probes, designed in sets containing a small number of mismatches, over a range of target concentrations. Probes by their nature have different sequence composition, and consequently different thermodynamic properties, but affinity differences alone do not explain all of the observed variation. In particular, rules for predicting probe response at one concentration may not be valid at another concentration. One way to demonstrate this is to consider the results presented in Figures 1 and 2, in which probes containing mismatches show binding differences from the perfect match probes only at some target concentrations. The most effective model of the observed responses over a range of target concentrations incorporates a multistate equilibrium model of hybridization, the PPB. The use of a range of target concentrations to collect observations was very important in establishing the general applicability of this model: not only did it point to a weakness in the free energy model but it reflects the experimental reality that not all genes or genomic segments are present at the same concentration in most microarray experiments.

### Detection of low numbers of mismatches in long oligonucleotides

Given the large number of possible locations and permutations for mismatches, it has been not feasible to study all of the types of mismatches that can occur in a 50-mer probe, and still give a measurable signal. In general, mismatches in the middle of a sequence affect the binding affinity most severely [18], [19], [20]. Hence the ten probe sets used here include single-, double- and triple-MM classes, with the mismatches introduced in the center. We used homomeric transversions ( i.e. A↔T, G↔C), which allows comparison to a number of studies based on the Affymetrix short-oligomer platform.

There are differences in trends seen with the shorter probes and these longer oligonucleotides. For example, the members of the class of probes with one and two mismatches show similar responses to one another regardless of the position or type of base change. The difference in stability is very small, an average of 5% less stability for single-MM than for the PM, and the platform is probably not sensitive enough to pick up differences. It is also notable that for none of the single-MM probes was the signal higher than for the cognate PM probe, while MM>PM signal is a fairly common observation on Affymetrix GeneChip arrays [44]. This may be due to the lack of complex background containing competing targets in our hybridization reactions, or it may be due to our use of an end-labeling protocol that is quantitative and has no sequence dependence. A phenomenon not noted in other literature is the existence of a sweet spot in the target concentration gradient, where perfect match-mismatch discrimination is possible for a number of classes. In our arrays this region is where target concentrations are in the range 100–200pM. This may provide useful insight in the analysis of SNP chip data, where the target concentration range is smaller than for expression arrays.

### Predicting microarray outcomes

Understanding the factors that affect microarray signal is of importance to both the engineers who design an array and the scientists who use it. The change in *ΔG* of the duplex is a factor that the array designer can incorporate, but we demonstrate that it is only good at predicting responses at relatively high concentrations of target. If the experimental design includes arrays that capture the response plateaus of probes, or if probe concentration information is provided by a particular supplier, then an analysis can use the probe percent bound (PPB) to obtain much more accurate estimates of target concentration. The PPB is an equilibrium model of systems of interacting oligonucleotides, and appears to be an excellent predictor of detected signal intensity on the microarray surface across many target concentrations, for the limited number of sequences we were able to test. A similar value, although calculated differently, was used to model the response of 25-mer probes to several target concentrations [45].

Despite studies showing that surfaces do affect hybridization kinetics in microarrays, it is generally assumed that solution models provide a reasonable approximation to probe-target interaction at the microarray surface [18], [35]. The apparent effectiveness of the PPB model, which arises from solution studies, supports the appropriateness of the solution model. It also reinforces the importance of incorporating biophysical factors into both the design and analysis of microarrays [19], [43], [44], [46], [47]. The limitation to the PPB calculation is its dependence on knowing either the response plateau for a probe or its concentration. However, for most commercial microarray platforms this quantity is either published or can be estimated; in some cases there are published measurements from independent sources (see [45] for an example). Otherwise the probe saturation concentration or the approximate probe concentration can be deduced by a simple target concentration titration experiment.

Our primary goal in this experiment was to measure and then accurately model the differences in response exhibited by perfectly matched duplexes and related mismatch duplexes. While remaining in probe excess we wanted to determine whether differences in input target concentration led to deviation from a simple affinity-based response model, and indeed such an effect was observed. We determined that *ΔG_duplex_* does not explain the differences in signal as effectively as does the probe percent bound (PPB). The correlation between *ΔG_duplex_* and the observed signal intensity increases as the target concentration increases (Fig. 6), in agreement with results presented by Li *et al.* [45] but it does not capture the concentration dependence of microarray hybridization reactions. It is important to note that, while less sensitive to the position and identity of a mismatch than short probes, long oligonucleotide probes have physical hybridization profiles that can be modeled with well-known solution parameters. Examining the fitted parameters of the Langmuir isotherm model for the four groups of probes (Fig. 4) confirms the similarities of their physical hybridization profile to that of shorter probes. Hekstra *et al.* [43] reported similar results when comparing the fitted parameters between PM and single-MM probes when the Langmuir isotherm model is applied to Affymetrix GeneChip probes. Physical models have clear advantages in the analysis of microarray data, as demonstrated by approaches like that developed by Abdueva *et al.* [46].

### Impact on array design and analysis

Target concentration is the principal unknown quantity in microarray applications. For gene expression microarrays it is also the goal of the experiment and cannot be known beforehand. For other types of arrays, like SNP arrays, allele calls are desired and target concentration ranges are much more restricted; controlled target input is possible and may be worth the extra effort, especially if it allows maximal discrimination of single-nucleotide mismatches. Default values for target concentration in many probe design pipelines that do take duplex stability into account are often relatively high, from 3–50 nM or even 1 µM [13], [48], [49]. This is probably misleading with respect to actual target concentrations and where responses will be most informative. We therefore recommend that expected target concentrations of 100 or 200 pM should be considered in biophysical modeling to support probe selection for glass-slide array platforms on which probe concentration is 10–20µM, like the ones used in this study. Where commercial platforms with high lot reproducibility have been used in experiments with either spike-ins or independent PCR validation, it should be possible to project target concentration from intensity across the experiment and calibrate responses over a range of concentrations. The PPB model might be of use here to obtain more accurate target concentration estimates from experiments using long-oligonucleotide arrays.

### Conclusion

In this experiment, we have demonstrated that single mismatches in long oligonucleotide probe/target pairs do not abolish signal, but that the signal is significantly lower than for the perfectly matched probe-target pair, at target concentrations in the linear range of the experiment. Additional mismatches result in measureable signal but progressively larger decreases in intensity. When mismatches are located in the three central positions in a 50-mer, the type of MM base and its position cannot be inferred from the intensity change. At all but the lowest concentrations of target (6.25pM and 12.5 pM), single, double and triple mismatches between the probe and target give detectable and significantly different responses from the PM-target pair.

The second significant finding is that binding predictions derived from multi-state solution hybridization models do an excellent job of predicting response characteristics for long-oligo microarrays over many concentrations. We show this to be true even though such models were developed for much shorter oligonucleotides in solution and have not previously been used to accurately model the behavior of oligonucleotides >40nt. While it remains to be seen whether this finding will hold for more complex probe-target systems, this study suggests that appropriately parameterized solution models of hybridization will accurately represent interactions on the oligonucleotide microarray surface even for non-ideal oligonucleotides. This suggests that probe designers and scientist performing transcriptomics experiments can use these modeling tools with confidence when selecting optimal probes and analyzing experimental results.

## Materials and Methods

### Probe design and selection

Perfect match probes (PM) were designed using a two-stage process in which sequence screening was followed by biophysical modeling (Figure S1). Briefly, Yoda [48] was the selection tool and probes were generated using the command shown in Figure S1. Probes were generated based on characterized gene sequences from *Brucella suis*. Yoda-generated candidate probes were then screened for secondary structure with *hybrid-ss-min*, from the UNAFold package [50] using 60°C as the folding temperature, Sodium concentration of 0.6 mol/L and DNA for the −n option (defaults for all the other parameters ). Candidates surviving this filter were passed into OMP, which predicts hybridization affinity of a perfect duplex.. The sequences of the final candidate pool were stored in a MySQL database; the ten optimal sequences probes were chosen using an *ad hoc* multi-criterion sort. Six mismatch (MM) counterparts were designated for each of the ten probes sequences. Mismatch sets included single base changes at positions 24,25 or 26, two-base changes at positions 24+25 or 25+26, and a 3-base change covering positions 24+25+26; this produces a set of 7 related sequences to compare for each of the ten base sequences. All changes were the homomeric transversions, A↔T and G↔C. Six sequences matching *Arabidopsis thaliana* genes were designed using the same approach, to serve as controls.

### Fabrication of microarray slides

All probes were synthesized with amino-C6 linkers at the 5′ end by Operon Biotechnologies (Huntsville, AL), and targets complementary to the perfect match sequences were synthesized with Cy3 attached. Microarray slides were manufactured by ArrayIt (Sunnyvale, CA). Each probe was spotted in six replicate spots on each slide. After preliminary hybridization tests the 10 µM probe solution was chosen as the optimal spotting concentration for the current experiment. All oligonucleotides were HPLC purified to ensure length and labeling uniformity.

### Preparation of target mixture

Target oligonucleotides were re-suspended to 100 µM concentration in 2× SSC and checked for correct concentration and integrity via spectrophotometry and polyacrylamide gel electrophoresis. A concentrated master mix was made containing equimolar concentrations of the targets, and aliquoted, with the aliquots frozen at −20C. For each hybridization experiment an aliquot of the master mix was diluted to the desired final concentration in hybridization buffer (0.4 mg/ml salmon sperm, 4× SSC, 0.5% SDS) which had been heated to 95°C for 5 minutes, and then chilled on ice for 10 minutes before the addition of the oligos. The following target concentrations were used: 5000 pM, 1000 pM, 200 pM, 100 pM, 50 pM, 25 pM, 12.5 pM and 6.25 pM.

### Array hybridization

The slides (two technical replicates for each concentration) were placed in an HS 4800 Pro Hybridization Station (Tecan, Mannedorf, Switzerland), which had been preheated to 55 °C. All wash solutions were also preheated by the hybridization station. The slides were then wetted by a brief rinse with a Hybridization Wash solution (0.5× SSC, 0.005% SDS) so that the slide was not dry when it received the blocking buffer. The slides were blocked with BlockIt solution (ArrayIt, Sunnyvale, CA) for 30 minutes. The slides were then washed again for 2 minutes with the Hybridization Wash solution. 60 µL of hybridization solution containing the target was then added and the slides were incubated for 18 hours at 55°C. Slides were subjected to mechanical agitation at medium intensity (1.1 minutes agitation with 3.5 minutes break) during hybridization. Then the slides were washed three times in the Hybridization Wash solution for 30 seconds, then washed for a minute with the Hybridization Wash solution and cooled to 50°C. The slides were then washed with TE buffer for 30 seconds and cooled to 45°C, washed with 0.5× TE buffer for 30 seconds and cooled to 40°C, washed with 5% ethanol (Sigma Aldrich, St. Louis, MO) for 1 minute and cooled to 30 °C, and finally washed twice with ddH_2_O for 40 seconds and cooled to 25 °C. After these washes the slide was dried under nitrogen for 3 minutes and scanned immediately.

### Image acquisition and data analysis

Slides were scanned with the 532nm laser, a 575nm filter, 10µm resolution, an over sampling factor of 2 and a 150 PMT gain in the LS Reloaded Scanner (Tecan, Mannedorf, Switzerland). Images were saved in the Tagged Image File format (tif) and then analyzed using SPOT (CSIRO, Sydney, Australia, http://www.hca-vision.com/product_spot.html), with the segmentation option set to ‘seeded region growing’. The quality of each array and its spots were determined according to He *et al.* [28]. The raw intensities were loaded into the LIMMA [51] package (version 2.12.0) of Bioconductor [52] using the *read.maimages* function. LIMMA was also used for between-array (quantile) normalization for each pair of technical replicates. The analysis presented in this work was done using R (version 2.6.1) [53].

### OMP hybridization simulation

Hybridization simulations for probe design and signal intensity prediction were done using OMP DE (version 1.1.0.2089) running on Red Hat Enterprise Linux 4. In this case, the complete system of 76 probes (including negative controls) and ten 50-mer targets could be simulated simultaneously, as the sequence lengths and number of probes and targets in this hybridization system was manageable by OMP in a reasonable period of time. All probe and target sequences are included in Table S1. For each probe, the following data were collected from the OMP output: *ΔG_duplex_*, Δ*H_duplex_*, Δ*S_duplex_*, *T_m_*, *basepair count* and *probe percent bound*. All the parameters used in the hybridization simulations are shown explicitly in Figure S1. Target concentrations for signal prediction are listed in the Preparation of Target Mixture section above.

### Langmuir isotherm fitting

The Langmuir isotherm is a chemical adsorption model [54] that has been applied successfully to short oligonucleotide microarrays [19], [43], [46]. The model is simply a hyperbolic response function in the form of:
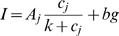
(1)where *I_j_* is the signal intensity from the probe at target concentration *j*. *A*, *K* and *bg* are the model fitting parameters, *c* is the target *j^th^* concentration in pM. This model has three free parameters (*A*, *K* and *bg*) fitted to eight different concentrations (5000 pM, 1000 pM, 200 pM, 100 pM, 50 pM, 25 pM, 12.5 pM and 6.25 pM). The fitting parameter *K* is the probe affinity constant, *A* is the saturation intensity (assuming no cross-hybridization, i.e. *bg* = 0) and *bg* is a background component [43], [45], [54], [55]. The model was fitted using the *nls* function of R (version 2.6.1) [53].

### Predicting signal intensity using probe percent bound

A simple linear model, with only two free parameters, was used to predict the signal intensity. It is based on the probe percent bound (PPB), following the equation below:
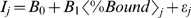
(2)where *I_j_* is the signal intensity from the probe at target concentration *j*. *%Bound* is the PPB of the probe at target concentration *j*. *B_0_* and *B_1_* are free parameters and *ε_j_* is an error term. OMP percent binding predictions were computed in the presence of all targets (competitive hybridization), at eight different target concentrations: 5000 pM, 1000 pM, 200 pM, 100 pM, 50 pM, 25 pM, 12.5 pM and 6.25 pM. This model has two free parameters (*B_0_* and *B_1_*) fitted to eight PPB values. The model was fitted using the *lm* function of R (version 2.6.1) [53].

### Code and data

The code and data used in this study are available as an R package and can be downloaded from http://bioinfo.uncc.edu/rgharaib/unccMM. The complete set of figures generated from the data produced, and referenced in this article, can be reproduced using this package.

## Supporting Information

Table S1 Probe set sequences
(1.24 MB TIF)

Figure S1 Probe design schema
(0.33 MB TIF)

Figure S2 (A) Relationship between probe signal intensity and predicted percent bound (PPB) at each target concentration for probe 5037 from the single-MM group. Points represent observed intensities, and the solid line represents the fit of the model (equation 2). (B) Box plots for the obtained R2 and p-values of the null hypothesis that the B1 parameter in equation 2 is equal to zero from all single-MM probes. Dotted line indicates P = 0.05.
(0.33 MB TIF)

Figure S3 (A) Relationship between probe signal intensity and predicted percent bound (PPB) at each target concentration for probe 5056 from the double-MM group. Points represent observed intensities, and the solid line represents the fit of the model (equation 2). (B) Box plots for the obtained R2 and p-values of the null hypothesis that the B1 parameter in equation 2 is equal to zero from all double-MM probes. Dotted line indicates P = 0.05.
(0.33 MB TIF)

Figure S4 (A) Relationship between probe signal intensity and predicted percent bound (PPB) at each target concentration for probe 5034 from the triple-MM group. Points represent observed intensities, and the solid line represents the fit of the model (equation 2). (B) Box plots for the obtained R2 and p-values of the null hypothesis that the B1 parameter in equation 2 is equal to zero from all triple-MM probes. Dotted line indicates P = 0.05.
(0.33 MB TIF)

Figure S5 Relationship between probe signal intensity and predicted percent bound (PPB) for all probes. Points represent observed intensities, and the solid line represents the fit of the model (equation 2).
(0.39 MB TIF)

Figure S6 Relationship between probe PPB values for PM (probe 5001) versus single-MM (5011, 5012 and 5013), double-MM (5014, 5015) and triple-MM (5016).
(0.33 MB TIF)

Figure S7 A) Relationship between probe PPB values for PM (5002) versus single-MM (5017, 5018 and 5019), double-MM (5020 and 5021) and triple-MM (5022); B) for PM (5003) versus single-MM (5023, 5024 and 5025), double-MM (5026 and 5027) and triple-MM (5028); C) for PM (5004) versus single-MM (5029, 5030 and 5031), double-MM (5032 and 5033) and triple-MM (5034); D) for PM (5005) versus single-MM (5035, 5036 and 5037), double-MM (5038 and 5039) and triple-MM (5040); E) for PM (5006) versus single-MM (5041, 5042 and 5043), double-MM (5044 and 5045) and triple-MM (5046). F); for PM (5007) versus single-MM (5047, 5048 and 5049), double-MM (5050 and 5051) and triple-MM (5052); G) for PM (5008) versus single-MM (5053, 5054 and 5055), double-MM (5056 and 5057) and triple-MM (5058); H) for PM (5009) versus single-MM (5059, 5060 and 5061), double-MM (5062 and 5063) and triple-MM (5064); I) for PM (5010) versus single-MM (5065, 5066 and 5067), double-MM (5068 and 5069) and triple-MM (5070).
(2.21 MB TIF)
