# Supplementary figures and images for: Application of Equilibrium Models of Solution Hybridization to Microarray Design and Analysis
Source: PLoS One. 2010 Jun 10;5(6):e11048. doi: 10.1371/journal.pone.0011048 (PMC2883574; doi:10.1371/journal.pone.0011048)

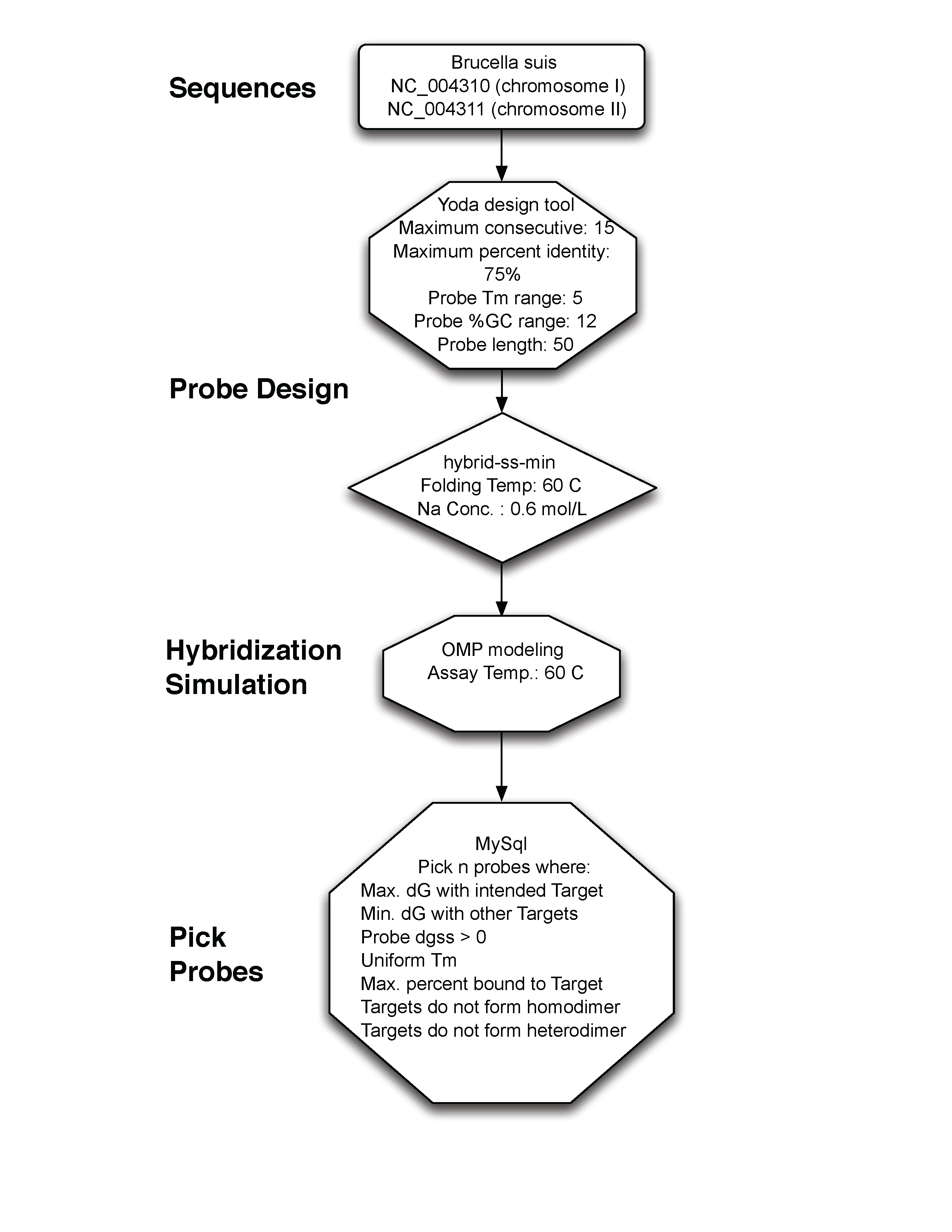

Supplement: Table S1 — Probe set sequences (1.24 MB TIF) [file pone.0011048.s001.tif]

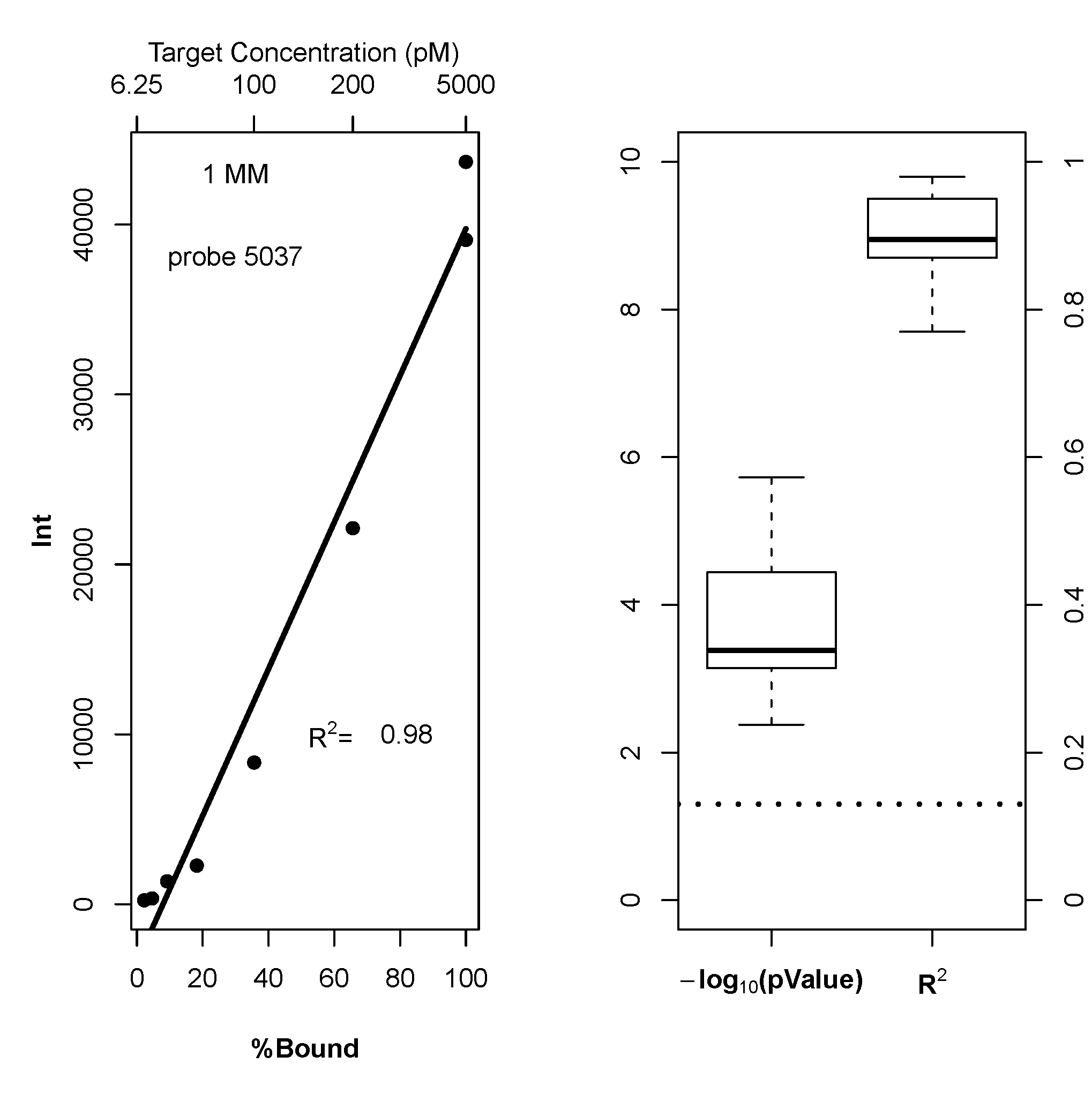

Supplement: Figure S1 — Probe design schema (0.33 MB TIF) [file pone.0011048.s002.tif]

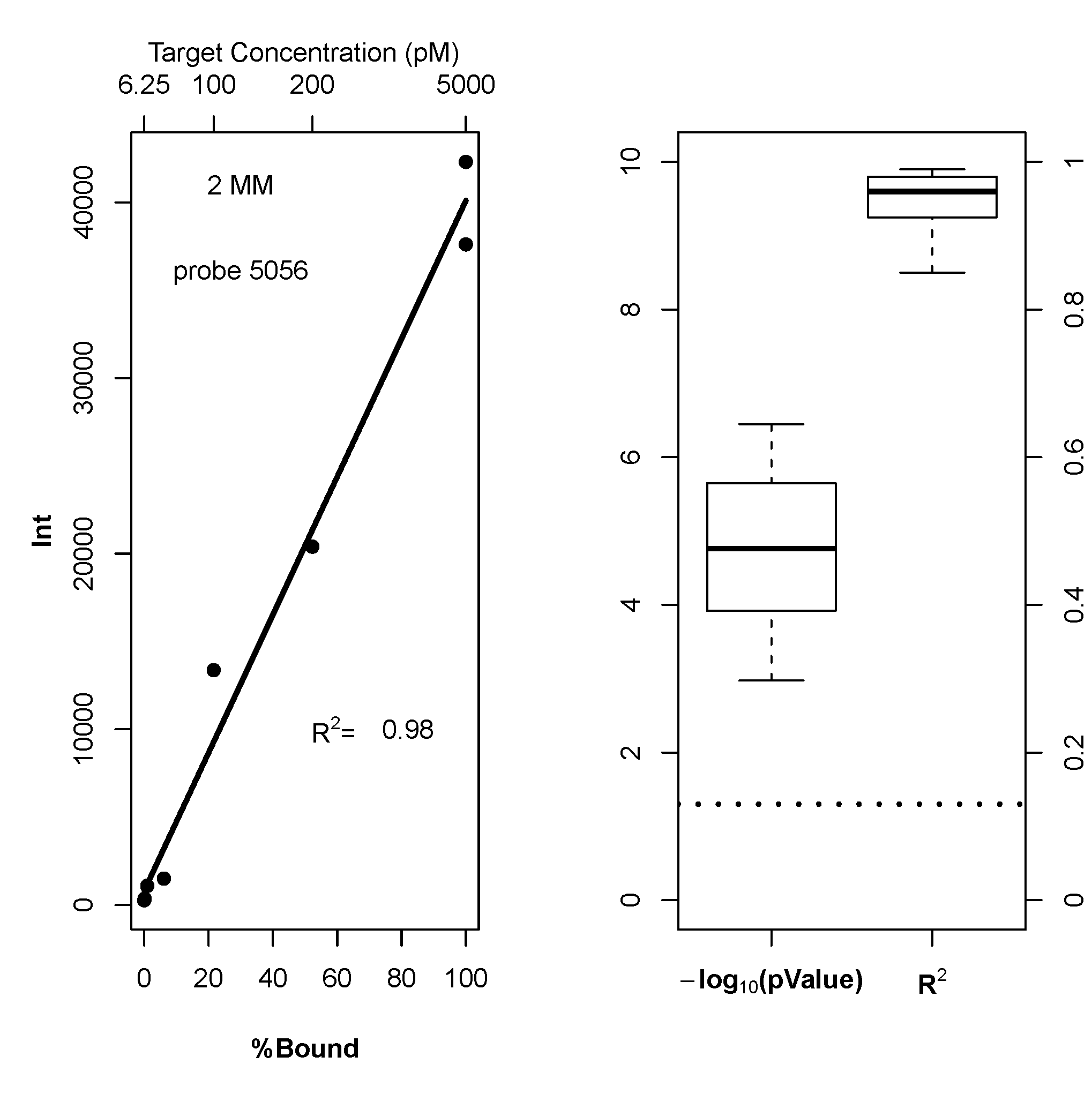

Supplement: Figure S2 — (A) Relationship between probe signal intensity and predicted percent bound (PPB) at each target concentration for probe 5037 from the single-MM group. Points represent observed intensities, and the solid line represents the fit of the model (equation 2). (B) Box plots for the obtained R2 and p-values of the null hypothesis that the B1 parameter in equation 2 is equal to zero from all single-MM probes. Dotted line indicates P = 0.05. (0.33 MB TIF) [file pone.0011048.s003.tif]

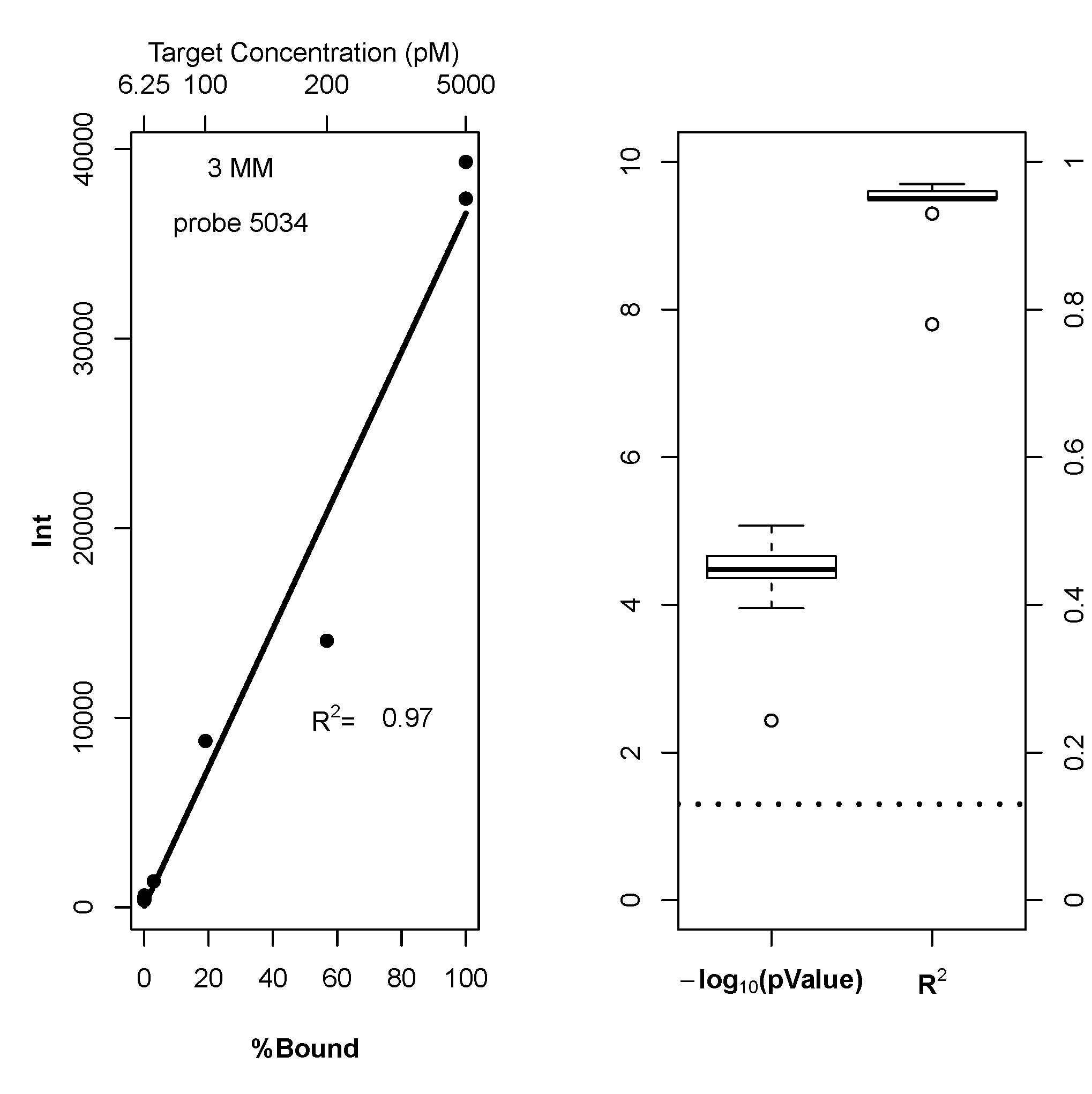

Supplement: Figure S3 — (A) Relationship between probe signal intensity and predicted percent bound (PPB) at each target concentration for probe 5056 from the double-MM group. Points represent observed intensities, and the solid line represents the fit of the model (equation 2). (B) Box plots for the obtained R2 and p-values of the null hypothesis that the B1 parameter in equation 2 is equal to zero from all double-MM probes. Dotted line indicates P = 0.05. (0.33 MB TIF) [file pone.0011048.s004.tif]

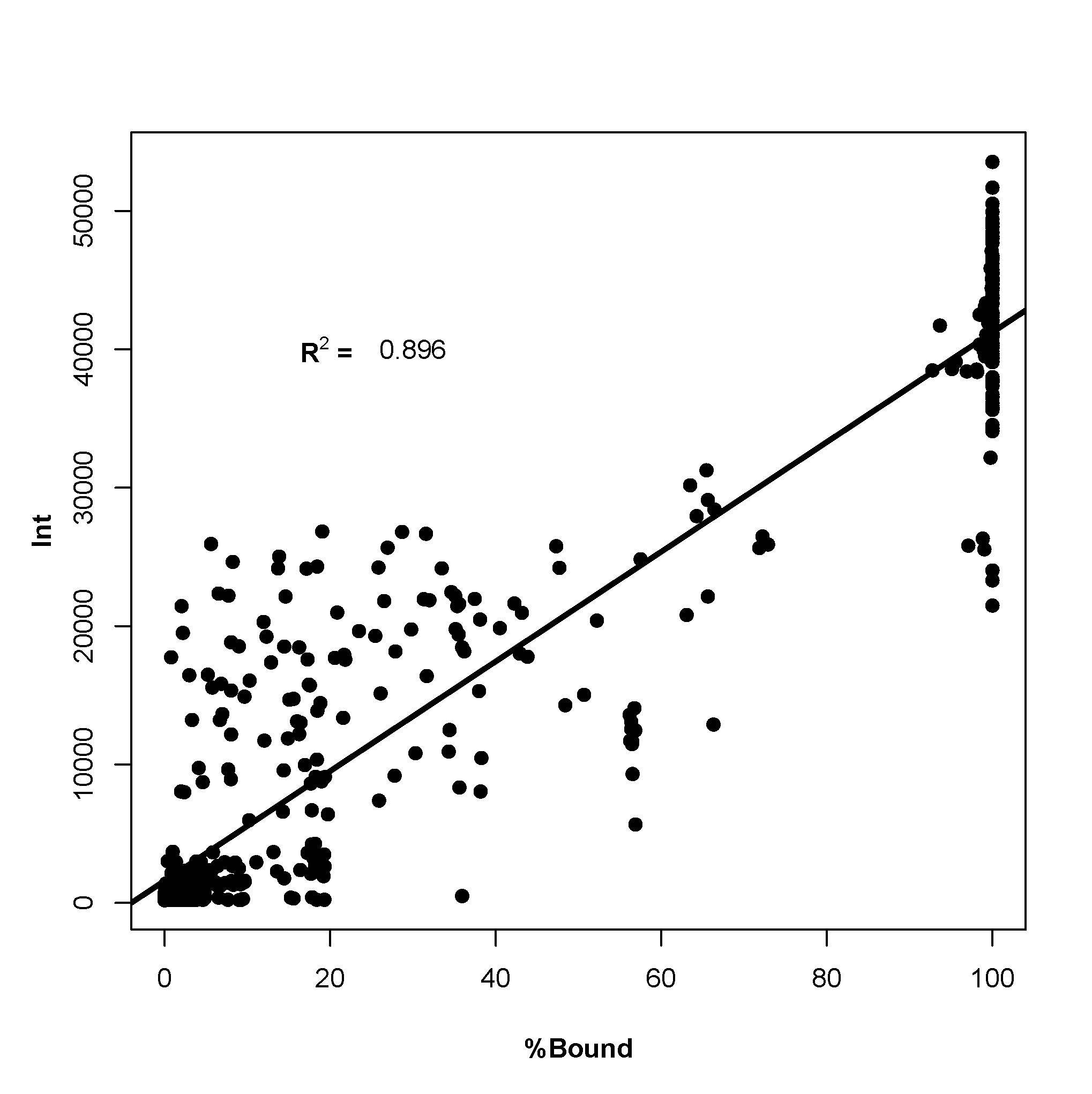

Supplement: Figure S4 — (A) Relationship between probe signal intensity and predicted percent bound (PPB) at each target concentration for probe 5034 from the triple-MM group. Points represent observed intensities, and the solid line represents the fit of the model (equation 2). (B) Box plots for the obtained R2 and p-values of the null hypothesis that the B1 parameter in equation 2 is equal to zero from all triple-MM probes. Dotted line indicates P = 0.05. (0.33 MB TIF) [file pone.0011048.s005.tif]

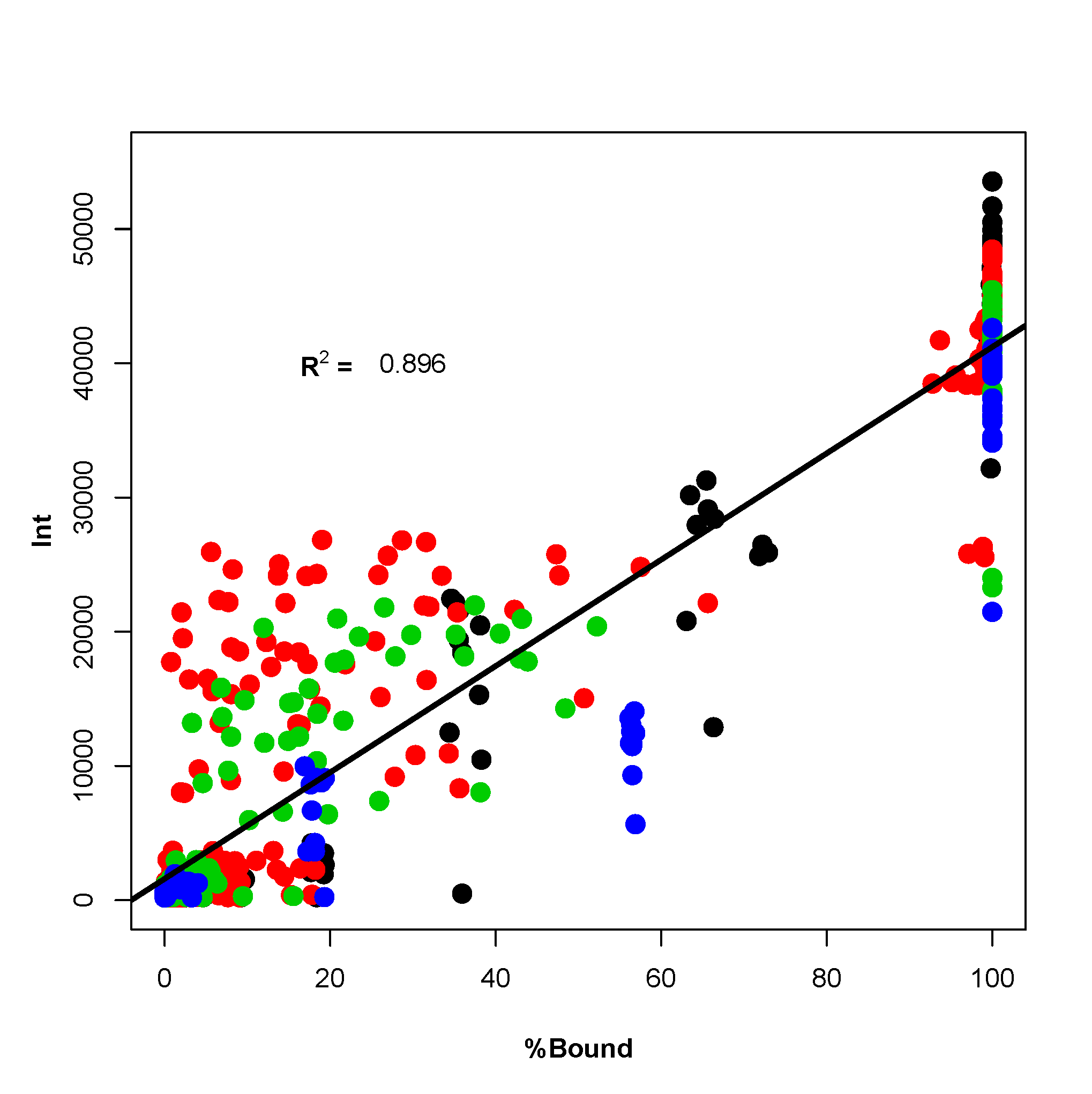

Supplement: Figure S5 — Relationship between probe signal intensity and predicted percent bound (PPB) for all probes. Points represent observed intensities, and the solid line represents the fit of the model (equation 2). (0.39 MB TIF) [file pone.0011048.s006.tif]

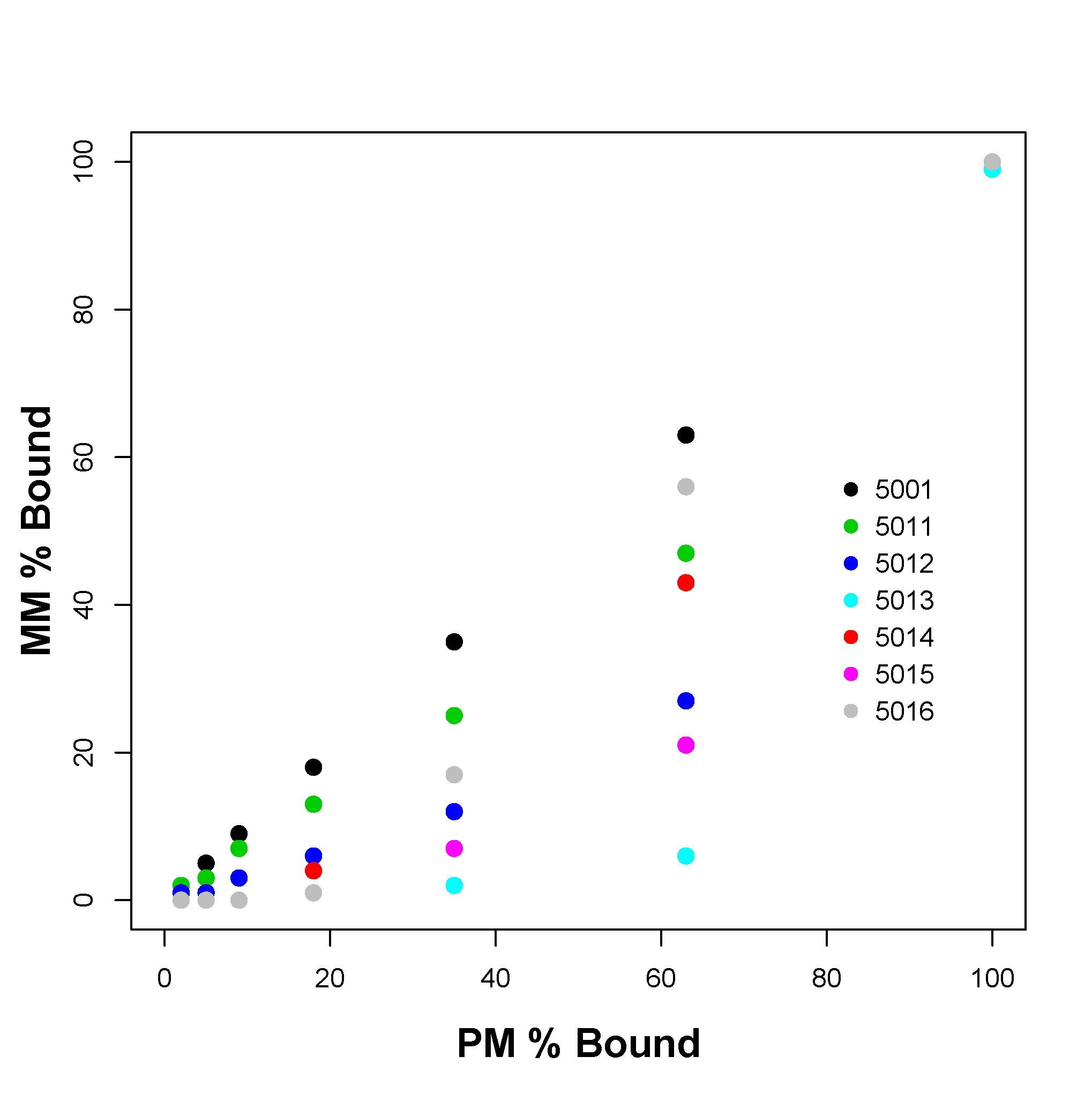

Supplement: Figure S6 — Relationship between probe PPB values for PM (probe 5001) versus single-MM (5011, 5012 and 5013), double-MM (5014, 5015) and triple-MM (5016). (0.33 MB TIF) [file pone.0011048.s007.tif]

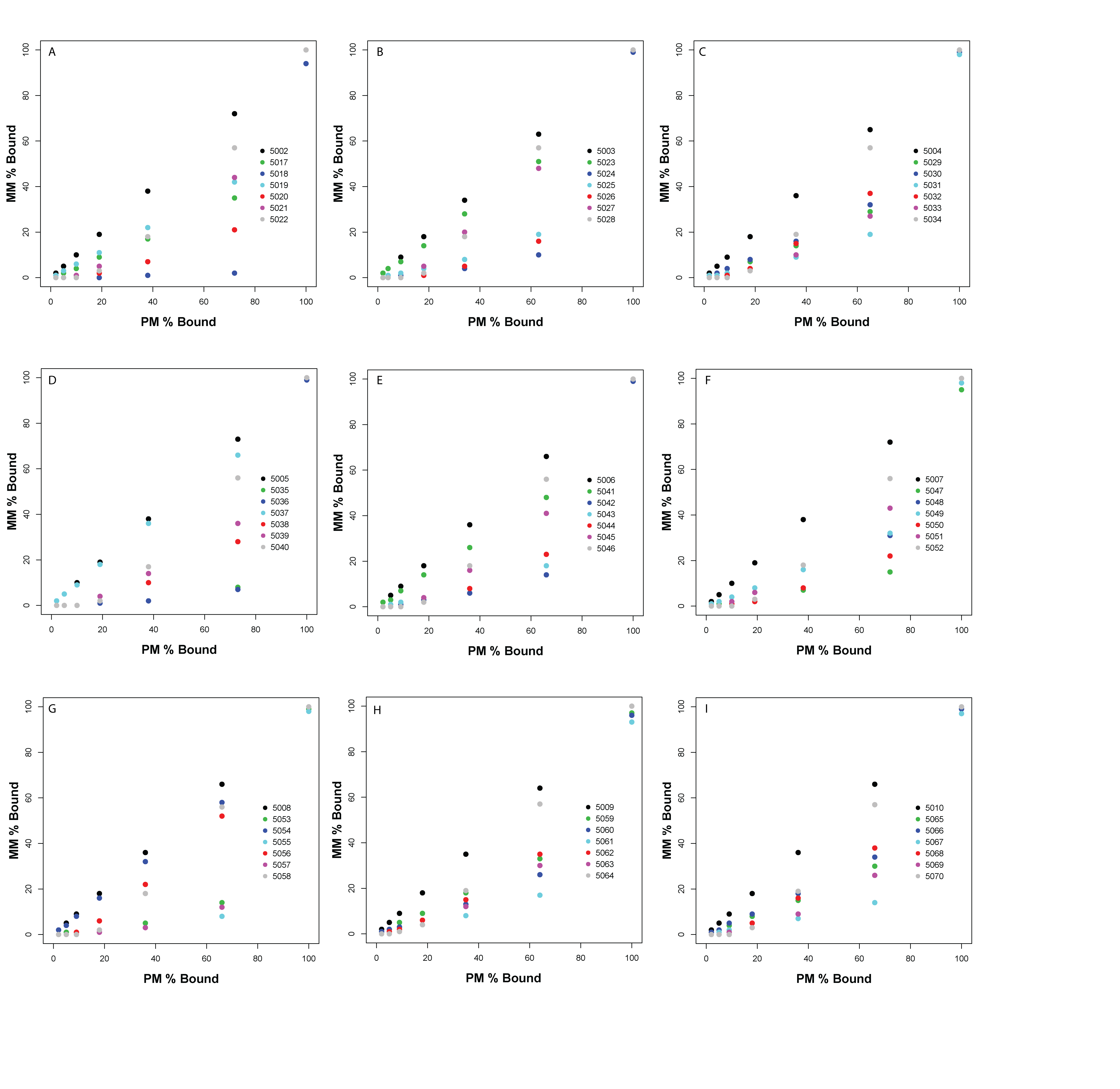

Supplement: Figure S7 — A) Relationship between probe PPB values for PM (5002) versus single-MM (5017, 5018 and 5019), double-MM (5020 and 5021) and triple-MM (5022); B) for PM (5003) versus single-MM (5023, 5024 and 5025), double-MM (5026 and 5027) and triple-MM (5028); C) for PM (5004) versus single-MM (5029, 5030 and 5031), double-MM (5032 and 5033) and triple-MM (5034); D) for PM (5005) versus single-MM (5035, 5036 and 5037), double-MM (5038 and 5039) and triple-MM (5040); E) for PM (5006) versus single-MM (5041, 5042 and 5043), double-MM (5044 and 5045) and triple-MM (5046). F); for PM (5007) versus single-MM (5047, 5048 and 5049), double-MM (5050 and 5051) and triple-MM (5052); G) for PM (5008) versus single-MM (5053, 5054 and 5055), double-MM (5056 and 5057) and triple-MM (5058); H) for PM (5009) versus single-MM (5059, 5060 and 5061), double-MM (5062 and 5063) and triple-MM (5064); I) for PM (5010) versus single-MM (5065, 5066 and 5067), double-MM (5068 and 5069) and triple-MM (5070). (2.21 MB TIF) [file pone.0011048.s008.tif]
